# Endocannabinoid receptor blockade reduces alanine aminotransferase in polycystic ovary syndrome independent of weight loss

**DOI:** 10.1186/s12902-017-0194-2

**Published:** 2017-07-14

**Authors:** Alison J. Dawson, Eric S. Kilpatrick, Anne-Marie Coady, Abeer M. M. Elshewehy, Youssra Dakroury, Lina Ahmed, Stephen L. Atkin, Thozhukat Sathyapalan

**Affiliations:** 1 Department of Diabetes and Endocrinology, University of Hull, Hull, UK; 2 Department of Clinical Biochemistry, Sidra Medical and Research Centre, Doha, Qatar; 3 Department of Obstetric Ultrasound, Hull & East Yorkshire Women’s & Children’s Hospital, Hull, UK; 4 Weill Cornell Medicine Qatar, Research Department, PO Box 24144, Doha, Qatar

**Keywords:** Alt, Rimonabant, Polycystic ovarian syndrome, Nafld

## Abstract

**Background:**

Evidence suggests that endocannabinoid system activation through the cannabinoid receptor 1 (CB1) is associated with enhanced liver injury, and CB1 antagonism may be beneficial. The aim of this study was to determine the impact of rimonabant (CB1 antagonist) on alanine aminotransferase (ALT), a hepatocellular injury marker, and a hepatic inflammatory cytokine profile.

**Methods:**

Post hoc review of 2 studies involving 50 obese women with PCOS and well matched for weight, randomised to weight reducing therapy; rimonabant (20 mg od) or orlistat (120 mg tds), or to insulin sensitising therapy metformin, (500 mg tds), or pioglitazone (45 mg od). No subject had non-alcoholic fatty liver disease (NAFLD).

**Results:**

Treatment with rimonabant for 12 weeks reduced both ALT and weight (*p* < 0.01), and there was a negative correlation between Δ ALT and Δ HOMA-IR (*p* < 0.001), but not between Δ ALT and Δ weight. There was a significant reduction of weight with orlistat (*p* < 0.01); however, orlistat, metformin and pioglitazone had no effect on ALT. The free androgen index fell in all groups (*p* < 0.05). The inflammatory marker hs-CRP was reduced by pioglitazone (*p* < 0.001) alone and did not correlate with changes in ALT. The inflammatory cytokine profile for IL-1β, IL-6, IL-7, IL-10, IL12, TNF-α, MCP-1 and INF-γ did not differ between groups. None of the interventions had an effect on biological variability of ALT.

**Conclusion:**

Rimonabant through CB1 receptor blockade decreased serum ALT that was independent of weight loss and hepatic inflammatory markers in obese women with PCOS without NAFLD.

**Trial registration:**

ISRCTN58369615 (February 2007; retrospectively registered) ISRCTN75758249 (October 2007; retrospectively registered).

## Background

It has been recently shown through in vitro and animal studies that the cannabinoid receptor 1 (CB-1) contributes to liver injury, inflammation and may be involved in the initiation of hepatocellular carcinoma through endocannabinoid system activation [1, 2]. Endogenous cannabinoids (EC) have been shown to be closely related to fatty liver metabolism [3, 4], modulating lipid metabolism and contributing to non-alcoholic fatty liver disease development (NAFLD) through [5] that may be ameliorated by CB1 receptor antagonism with antagonists such as rimonabant [5]. This is of particular importance given that NAFLD is the most common cause of chronic liver disease [6] leading to cirrhosis [7]. Liver enzymes such as alanine aminotransferase (ALT) are commonly used as a serum markers for liver function and may or may not be elevated in NAFLD that can only be confirmed by a liver biopsy, as the.

NAFLD has been reported to be of an increased prevalence in women with polycystic ovarian syndrome (PCOS), a condition that is the most common endocrine problem in premenopausal women [6]. Insulin resistance is common in the pathophysiology of PCOS that mechanistically increases hepatic stress and liver damage by increasing fatty acid oxidation [7]. Metformin reduces insulin resistance in PCOS [8] and has been shown to have some benefit in NAFLD [9]. Pioglitazone, another insulin sensitising agent, has also been shown to be beneficial in both PCOS and NAFLD [10, 11]. Medications causing weight loss (orlistat and rimonabant) have also been effective treatments in PCOS [10, 12] and orlistat has shown to improve features of NAFLD [13].

Further clinical studies to investigate the hepato-protective effect of rimonabant are not possible as the drug was withdrawn from the market because of neuropsychiatric side effects. We hypothesized that direct CB1 antagonism by rimonabant treatment would decrease ALT levels in subjects without NAFLD, would decrease biological variability of ALT and that this would be independent of weight loss. To investigate this we pooled 2 randomised trials focusing on the modulation of insulin resistance in women with PCOS without NAFLD.

## Methods

We performed a post hoc data analysis on 2 studies performed involving 50 subjects (Clinical trial registrations ISRCTN75758249, retrospectively registered and ISRCTN58369615, retrospectively registered [10, 12]). The first was a randomized open-label parallel study with metformin and rimonabant in 20 patients with PCOS with a body mass index (BMI) ≥30 kg/m2. The 20 patients were randomized to either metformin 500 mg t.d.s. or rimonabant 20 mg daily. The second study was a randomized open-label parallel study with metformin, pioglitazone and orlistat in 30 patients with PCOS with a body mass index (BMI) ≥30 kg/m2. The 30 patients were randomized comparing treatment with metformin (500 mg three times a day), orlistat (120 mg three times a day) and pioglitazone (45 mg once daily) in hyperandrogenic, anovulatory Caucasian women with PCOS. It can be seen that the four groups were weight matched with a BMI range of 34.2 to 41.0. In both cases the diagnosis of PCOS was based on all three diagnostic criteria of the Rotterdam consensus, namely clinical and biochemical evidence of hyperandrogenaemia (Ferriman–Gallwey score > 8 and FAI > 4, respectively), oligomenorrhoea or amenorrhoea, and polycystic ovaries on transvaginal ultrasound [10, 12]. As detailed all subjects had a normal liver ultrasound with no evidence of a fatty liver indicative of NAFLD. Subjects had no concurrent illness, were not on any medication for the preceding 6 months and were not planning to conceive. None of the patients had had a successful pregnancy or a miscarriage at least 5 years prior to the study entry. Subjects were advised not to change their lifestyle, including physical activity and dietary habits, during the study period. Non-classical 21-hydroxylase deficiency, hyperprolactinaemia, Cushing’s disease and androgen-secreting tumours were excluded by appropriate tests. Adherence was monitored by counting the returned medication. All patients gave written informed consent. Additional IRB approval was not needed for this post hoc analysis as informed consent from the patients for future analyses on anonymous data had been given at the time of the initial consent procedure.

Randomization was performed using a random number generator. Both studies were approved by the Hull and East Riding Local Research Ethics Committee and both studies have been conducted in accord with the Consolidated Standards for Reporting Trials (CONSORT) 2010 statement [14].

Clinical and biochemical assessments were performed at randomization and at the end of the 3-month period for the first study [10, 12]. For the second biological variability study, clinical and biochemical assessments were performed at each visit on 10 consecutive occasions at 4-day intervals before and 12-weeks after treatment [12].

Methods have been detailed previously [10, 12] but briefly, study bloods and measurements were undertaken after an overnight fast. Fasting venous blood was collected into serum gel and fluoride oxalate tubes. Samples were separated by centrifugation at 2000 g for 15 min at 4oC, and the aliquots stored at –20oC. Serum testosterone was measured on an Architect analyser (Abbott Laboratories, Maidenhead, UK) and SHBG was measured by immunometric assay with fluorescence detection on a DPC Immulite 2000 analyser (Euro/DPC, Llanberis, UK) using the manufacturer’s recommended protocol. The FAI was obtained as the total testosterone × 100/SHBG. Total cholesterol, triglycerides and high-density lipoprotein cholesterol (HDL-C) levels were measured enzymatically using a Synchron LX20 analyser (Beckman-Coulter, High Wycombe, UK). Low-density lipoprotein cholesterol (LDL-C) was calculated using the Friedewald equation. Serum insulin was assayed using a competitive chemiluminescent immunoassay performed on the manufacturer’s DPC Immulite 2000 analyser (Euro/DPC). The analytical sensitivity of the insulin assay was 2μU/ml, the coefficient of variation was 6%, and there was no stated cross-reactivity with proinsulin. Plasma glucose was measured using the Synchron LX20 analyser (Beckman-Coulter), using the manufacturer’s recommended protocol. The coefficient of variation for the assay was 1·2% at a mean glucose value of 94·6 mg/dl (5·3 mmol/l) during the study period. The insulin resistance (IR) was calculated using the homeostasis model assessment (HOMA) method [HOMA-IR = (insulin x glucose)/22·5].

The Bio-Plex 200 system with HTF (Bio-Rad, Hercules, CA) was used to evaluate the sera and the levels of IL-1β, IL-6, IL-7, IL-10, IL12, TNF-α, MCP-1 and INF-γ and compared to a set of standards that were run simultaneously in the assay [15].

ALT was used specifically in this study to calculate the HAIR score (hypertension, ALT and insulin resistance) that is a predictive model for NASH (area under the receiver operator curve of 0.85) [16, 17]. Given that the mean ALT may not differ, but the biological variability may decrease specifically with weight loss, ALT biological variation was determined by ALT measurement performed at each of 10 consecutive visits at 4-day intervals, before and after 12 weeks treatment. All samples were taken between 8 and 9 am. Clinical assessments were also taken at these visits. Before analysis the samples were thawed thoroughly and mixed. Alanine aminotransferase was measured on the Unicel® DxC 80 analyser (Beckamn-Coulter, High Wycome, UK). Data are reported as mean ± SD. AST and gammaGT were not measured but have not been shown to be any better than ALT in NAFLD.

### Statistical analysis

Statistical analysis was performed using SPSS for Windows version 16.0 (SPSS inc). The power of the studies was based on a significant reduction in total testosterone concentration after treatment with metformin [10, 12]. In data where distribution between individuals violated the assumptions of normality when tested using the Kolmogorov-Smirnov test, median values were used and differences calculated by paired Wilcoxon method. Given that these were randomised clinical trials baseline values were not compared or adjusted for [18]. Biovariability data was analysed by calculating analytical, within subject, and between subject variances (SD_A_^2^, SD_I_^2^, SD_G_^2^, respectively) as detailed previously (5,6). Using this technique, analytical variance (SD_A_^2^) was calculated from the difference between duplicate results for each specimen (SD_A_^2^ = Σd^2^/2N, where d is the difference between duplicates, and N is the number of paired results). The variance of the first set of duplicate results for each subject on the ten assessment days was used to calculate the average biological intraindividual variance (SD_I_^2^) by subtraction of SD_A_^2^ from the observed dispersion (equal to SD_I_^2^ + SD_A_^2^). Subtracting SD_I_^2^ + SD_A_^2^ from the overall variance of the set of first results determined the interindividual variance (SD_G_^2^). The intraindividual (SD_I_) and interindividual (SD_G_) variations were estimated as square roots of the respective variance component estimates. For all analysis, a two-tailed *P* < 0.05 was considered to indicate statistical significance.

## Results

The patients were weight matched in each group (Table 1) and no patient had a fatty liver on ultrasound or a positive HAIR score (data not shown). Within group analysis showed that rimonabant reduced mean ALT from 30.8 U/l to 24.4 U/l, *p* < 0.01. There was a significant weight loss from (104.6 vs. 98.4 kg; *p* < 0.01) and reduction in body mass index (BMI) (36.9 vs. 34.5 kg/m^2^; *p* < 0.01) in the rimonabant group. As previously reported, insulin resistance improved in the group treated with rimonabant (calculated by HOMA-IR method) *p* < 0.05 [12], and there was a correlation between HOMA and Δ ALT *r* = 0.639, *p* < 0.04. There was no correlation between Δ ALT and weight or BMI in the patients treated with rimonabant.

**Table 1:** Anthropometric and metabolic data

Parameter	Metformin *n* = 20		*P*-value	Rimonabant *n* = 10		*P*-value	Orlistat *n* = 10		*P*-value	Pioglitazone *n* = 10		*P*-value
	Baseline	12 weeks		Baseline	12 weeks		Baseline	12 weeks		Baseline	12 weeks	
Weight (kg)	103.8 ± 3.9	102.2 ± 4.1	0.08	104.6 ± 4.6	98.4 ± 4.7	<0.01	103.1 ± 6.9	97.1 ± 6.1	<0.01	96.3 ± 4.9	99.2 ± 5.1	0.06
BMI (kg/m^2^)	35.7 ± 1.4	35.09 ± 1.5	0.08	36.86 ± 1.0	34.52 ± 1.0	<0.01	37.4 ± 2.7	35.2 ± 2.4	<0.01	36.2 ± 1.8	37.3 ± 1.8	0.06
FAI (%)	20.5 ± 4.0	18.2 ± 3.0	0.04	26.6 ± 6.1	16.6 ± 4.1	<0.01	16.4 ± 2.4	12.0 ± 2.4	0.01	18.1 ± 2.9	12.7 ± 1.6	0.02
ALT (U/L)	20.5 ± 2.54	18.1 ± 1.93	0.14	30.8 ± 5.1	26.4 ± 6.0	<0.01	13.2 ± 2.0	12.5 ± 1.6	0.49	20.4 ± 7.0	18.6 ± 3.0	0.58
HOMA-IR	3.4 ± 0.7	3.4 ± 0.8	0.77	4.4 ± 0.5	3.4 ± 0.4	0.05	5.0 ± 0.8	3.7 ± 0.5	0.01	4.5 ± 0.8	2.5 ± 0.4	<0.01
hsCRP (mg/l)	4.2 ± 1.7	4.0 ± 1.3	0.9	5.6 ± 1.2	5.2 ± 1.2	0.7	6.2 ± 2.0	5.6 ± 2.0	0.6	7.2 ± 1.8	3.1 ± 0.8	<0.01
TC	6.0 ± 0.3	5.6±	0.12	5.1 ± 0.3	4.7 ± 0.3	0.19	5.1 ± 1.2	4.6 ± 0.2	0.6	5.0 ± 0.4	5.1 ± 0.3	0.9
LDL-C	3.9 ± 0.3	3.5 ± 0.3	0.06	3.3 ± 0.3	3.1 ± 0.3	0.21	3.1 ± 0.2	2.7 ± 0.2	0.08	3.1 ± 0.3	3.2 ± 0.4	0.8
HDL-C	1.2 ± 0.2	1.2 ± 0.1	0.7	1.2 ± 0.2	1.1 ± 0.1	0.4	1.2 ± 0.05	1.2 ± 0.03	0.9	1.2 ± 0.06	1.4 ± 0.1	0.8
TG	2.0 ± 0.3	1.9 ± 0.4	0.3	1.4 ± 0.2	1.3 ± 0.1	0.4	1.8 ± 0.2	1.7 ± 0.2	0.8	1.4 ± 0.2	1.2 ± 0.2	0/8

*BMI* body mass index, *FAI* free androgen index, *ALT* alanine aminotransferase, *HOMA-IR* homeostasis model assessment of insulin resistance, *hsCRP* high-sensitivity C-reactive protein

Data are presented as mean ± SD. All serum results are obtained from fasting variables. All variables were normally distributed. To convert values for glucose to milligrams per decilitre, divide by 0·056. To convert values for insulin to picomoles per litre, multiply by 6. To convert values for cholesterol to milligrams per decilitre, divide by 0·0259. To convert values for triglycerides to milligrams per decilitre, divide by 0·0113. To convert values for testosterone to nanograms per decilitre, divide by 0·03467. To convert values for SHBG to micrograms per decilitre, divide by 34·7. *TC* Total cholesterol, LDL-C, LDL-cholesterol, *HDL-C*, *HDL* cholesterol, *TG*, triglycerides *FAI* free androgen index

Neither metformin nor pioglitazone affected Δ ALT, weight or BMI (Table 1). Orlistat did not significantly reduce Δ ALT despite a reduction in their BMI (37·4 ± 2·7 vs. 35·2 ± 2·4) that represented a − 5·7% change (0·8), *p* < 0.01 after treatment (Table 1). The magnitude of weight loss did not differ between rimonabant or orlistat treatment.

The percentage change from baseline ALT for Rimonabant, Metformin, Orlistat and Pioglitazone were 21%, 12%, 8% and 9% respectively. ANOVA of the percentage change in ALT for Rimonabant, Metformin, Pioglitazone and Orlistat with Tukey’s post hoc analysis showed a significant reduction in ALT with Rimonabant therapy (*p* < 0.05) alone.

The FAI decreased in all groups with treatment (*p* < 0.05); ANOVA showed no difference between groups and the FAI did not correlate with ALT.

The intraindividual variation of ALT did not differ before or after rimonabant or metformin treatment both within and between groups (*p* > 0.05). Neither orlistat nor pioglitazone had any effect on the biological variability of ALT.

The inflammatory marker hs-CRP was significantly decreased by pioglitazone alone (*p* < 0.01) but not correlate with ALT change. The pro-inflammatory cytokine profile performed between the metformin and rimonabant groups: IL-1β, IL-6, IL-7, IL-10, IL12, TNF-α, MCP-1 and INF-γ did not differ following either treatment (Table 2).

**Table 2:** Comparison of the pro-inflammatory cytokine profile before and after 12 weeks treatment with metformin or rimonabant

Parameter	Metformin group (*n* = 10)			Rimonabant group (*n* = 10)		
	Baseline	12 weeks	*P-*value	Baseline	12 weeks	*P*-value
IL-1β	86 ± 56	105 ± 89	0.17	85 ± 77	91 ± 91	0.32
IL-6	12 ± 6	13 ± 7	0.31	17 ± 8	18 ± 9	0.31
IL-7	249 ± 18	252 ± 31	0.69	235 ± 47	263 ± 49	0.11
IL-10	23 *±* 32	27 *±* 30	0.29	10 *±* 6	10 *±* 6	0.92
IL-12	203 *±* 60	223 *±* 83	0.11	201 *±* 41	207 *±* 33	0.50
TNF-α	7 *±* 3	8 *±* 4	0.33	9 *±* 4	9 *±* 4	0.99
MCP-1	294 *±* 66	298 *±* 88	0.80	279 *±* 60	307 *±* 58	0.6
INF-γ	30 *±* 10	30 *±* 9	0.83	30 *±* 7	36 *±* 9	0.6

No difference in the inflammatory cytokine profile between metformin and rimonabant treated patients

*IL* interleukin, *TNF* tumour necrosis factor, *MCP* monocyte chemotactic protein, *INF* interferon

There were no changes in the lipid parameters for each of the treatment arms (Table 1).

No patient suffered any neuropsychatric symptoms during rimonabant therapy.

## Discussion

These data show that rimonabant reduced the ALT of obese PCOS women while orlistat also led to a significant weight reduction, there was no corresponding fall in ALT suggesting that the effect of rimonabant on ALT was independent of the weight loss effect, and this change in ALT correlated with a reduction in insulin resistance. However, there was a reduction in weight and a reduction insulin resistance for both rimonabant and orlistat therefore it is not clear if their insulin resistance was reduced by rimonabant through CB1 or indirectly through weight loss, though in an animal model rimonabant countered age-induced insulin resistance [19]. An adverse metabolic profile associated with hyperandrogenemia has been correlated to NAFLD in PCOS [20], however whilst all treatments reduced the FAI there was no correlation to changes in ALT, suggesting a hepatic dependent mechanism, and suggesting that the changes seen for rimonabant were independent of its affect on androgen reduction. Similarly, the reduction of the inflammatory marker hsCRP was only seen for pioglitazone and therefore likely modulated by peroxisome proliferator-activated receptor gamma, rather through potential CB1 blockade. There were no changes in the pre-inflammatory hepatic cytokine profile between metformin and rimonabant.

The EC system comprises of CB1 (found mainly in the brain and the peripheral tissues) and CB2 receptors found mainly in the immune and endothelial system [21]. The liver has a low expression of the EC receptor that subsequently becomes up-regulated following liver injury [22]. Recent in vitro and animal data indicates that activation of the EC system through CB1 may enhance liver damage with inflammation and hepatocellular carcinoma initiation [1, 2]. thus is a novel mediator of liver disease [23]. EC through the CB1 receptors [24] are closely related to fatty liver metabolism [3, 4] and associated with NAFLD by modulating lipid metabolism [5]. These results appear discordant to the large population ADAGIO-lipids trial that showed weight loss and a decrease in ALT with rimonabant [25], and another trial showing weight loss was associated with a reduction in liver fat [26]; however, the first was a specific population with an atherogenic lipid profile and established fatty liver infiltration and the second trial was specifically in metabolic syndrome patients with demonstrable increased liver fat, and neither population was representative of the PCOS subjects here who had a normal liver ultrasound.

Rimonabant, is an N-acylaminopiperidinyl derivative and was the first approved CB1 antagonist for the treatment of obesity. However, it was withdrawn from the market due an increase in psychiatric disorders [21]. Animal studies have shown that rimonabant treatment reduce CB1 expression in diet induced obese mice [3, 27] and treatment results in a decrease in steatosis and associated metabolic diseases [28]. In four large human trials, rimonabant reduced weight and reduced liver steatosis and insulin sensitivity [29–31], data in accord with the reduction in ALT seen in this study. ALT did not correlate with weight loss and no change was seen in ALT with weight loss due to orlistat therapy confirming that this was more likely a direct effect of rimonabant rather than an indirect effect through weight loss.

Whilst metformin [32] pioglitazone [9] have been reported to reduce ALT and improve NAFLD neither would have been expected to have an affect on the ALT in normal individuals as found here.

The biological variation of insulin resistance has been demonstrated to be increased in PCOS women compared to controls [33]. If ALT variability was related directly to fluctuation in insulin resistance then it may have been expected that there would have been a reduction with intervention [10]. That the biological variation of ALT was unchanged here suggests it is not dependent on insulin resistance and that other factors, as yet unknown, affect it more. In both studies, metformin did not result in any change in insulin resistance whereas rimonobant, pioglitazone and orlistat all resulted in an improvement in insulin sensitivity; however, as we have noted before the insulin sensitising action of metformin may be lost or reduced as BMI increases [10, 12].

Whilst a total of 50 subjects were included, this is still a small sample for a parallel study, though such a study with rimonabant is no longer possible to conduct. The inclusion of a weight matched normal population would have ideally also been included for each intervention. However, for the reduction in ALT with rimonabant not to be significant, all three other treatments would need to have actively increased ALT.

## Conclusion

Rimonabant reduced ALT in obese women with PCOS without liver disease independent of weight loss and inflammatory hepatic markers, and the fall in mean ALT correlated to the reduction of insulin resistance, though biological variability was unchanged, data in accord with the increasing evidence from animal and in vitro data of CB1 antagonist benefit in liver disease.

## Abbreviations

ALT: Alanine aminotransferase
BMI: Body mass index
CB1: Cannabinoid receptor 1
EC: Endogenous cannabinoids
FAI: Free androgen index
HOMA-IR: Insulin resistance
hsCRP: C reactive protein
IL: Interleukin
INF: Interferon
MCP: Monocyte chemotactic protein
NAFLD: Non alcoholic fatty liver disease
PCOS: Polycystic ovary syndrome
TNF: Tumour necrosis factor.
